# Supplementary material for: Complexome profiling of the Chlamydomonas psb28 mutant reveals TEF5 as an early PSII assembly factor
Source: Plant Cell. 2025 Mar 17;37(6):koaf055. doi: 10.1093/plcell/koaf055 (PMC12164590; doi:10.1093/plcell/koaf055)
Supplement: koaf055_Supplementary_Data [file koaf055_supplementary_data.zip › psb28_interactive.zip!/plots/intensity/qualityPlots/peptides/TEF6_Cre16.g665250.t1.2.html]

### Cre16.g665250.t1.2

Trivial name: TEF6  
SeqCoverage [median]: 38.80  
Mass: 29.63 kDa  
  
 

| Sequence | MissedCleavage | Mass [Da] | wtIntensity | muIntensity | Score |
| --- | --- | --- | --- | --- | --- |
| ASAEPSAPAKPAGSQEYVEDSEFAVSK | 1 | 2751.29 | 7673.46 | 19267.83 | 110.03 |
| DKKDVLPPLLPGLK | 2 | 1531.93 | 632.27 | 886.10 | 49.31 |
| DVLPPLLPGLK | 0 | 1160.72 | 15147.05 | 52201.40 | 90.60 |
| ESQMTDIQK | 0 | 1078.50 | 31466.40 | 68884.40 | 260.23 |
| ESQMTDIQKQIR | 1 | 1475.74 | 0.00 | 831.25 | 92.92 |
| FLPTLIGIR | 0 | 1028.64 | 0.00 | 5671.33 | 82.28 |
| FRYGDEQHLEEALDR | 1 | 1876.87 | 6422.09 | 36580.63 | 131.24 |
| IFKPTRPGGVAR | 2 | 1297.76 | 3538.64 | 9590.99 | 72.65 |
| IQSFFGPGIVATVTK | 0 | 1563.87 | 12510.40 | 44653.40 | 204.94 |
| KDVLPPLLPGLK | 1 | 1288.81 | 433.73 | 0.00 | 43.38 |
| PAGSQEYVEDSEFAVSK | 0 | 1841.83 | 0.00 | 765.48 | 136.24 |
| SGKDKKDVLPPLLPGLK | 3 | 1804.08 | 1339.37 | 13304.64 | 58.83 |
| YGDEQHLEEALDR | 0 | 1573.70 | 7070.77 | 30698.14 | 159.12 |
|  |  |  | 86234.2 | 283335.6 |  |
